# Effects of age, sex, and axial length on macular structure and perfusion in normal eyes

**DOI:** 10.1097/MD.0000000000049020

**Published:** 2026-05-29

**Authors:** Yuki Ito, Hiroaki Endo, Satoru Kase, Natsuko Shichinohe, Mitsuo Takahashi, Manabu Kase

**Affiliations:** a Department of Ophthalmology, Teine Keijinkai Hospital, Sapporo, Japan; b Department of Ophthalmology, Faculty of Medicine and Graduate School of Medicine, Hokkaido University, Sapporo, Japan; c Department of Ophthalmology, Nara Medical University School of Medicine, Kashihara, Japan; d Teine Yokoi Eye Clinic, Sapporo, Japan.

**Keywords:** axial length, macular structure, optical coherence tomography angiography, perfusion density, retinal thickness

## Abstract

This study aimed to investigate how age, sex, refractive error, and axial length are related to macular structure and perfusion in normal eyes. This retrospective, cross-sectional study included 297 eyes of 297 patients without ocular diseases other than cataract who presented to Teine Keijinkai Hospital. All subjects underwent optical coherence tomography (OCT) and OCT angiography. Multiple regression analysis was performed with retinal thickness and perfusion density in the 9 Early Treatment Diabetic Retinopathy Study subfields as dependent variables, and age, sex, refractive error, and axial length as independent variables. Results showed that older age was associated with decreased retinal thickness in most regions excluding the central region, and decreased perfusion density in all regions (*P* < .05). Females had significantly thinner retinas in all regions (*P* < .05) but showed no significant difference in perfusion density. Longer axial length was associated with increased retinal thickness in the central region (*P* < .05), whereas it was linked to decreased values for both thickness and perfusion density in the outer regions (*P* < .05). Refractive error was not correlated with either parameter. In conclusion, retinal thickness and perfusion density in normal eyes are significantly associated with age, sex, and axial length. Considering these physiological factors is essential for the accurate interpretation of OCT and OCT angiography data in clinical practice.

## 1. Introduction

Optical coherence tomography (OCT) and OCT angiography (OCTA) have revolutionized the noninvasive assessment of macular structure and vasculature. Previous studies worldwide have demonstrated that macular parameters are influenced by various physiological factors, although findings remain partially inconsistent. For instance, while age-related macular thinning is frequently reported, the effect of aging on foveal thickness remains debated.^[1–4]^ Similarly, age-related reductions in capillary density have been documented,^[5–7]^ but sex-related differences in vascularity require further investigation due to conflicting results.^[8–11]^ Furthermore, in high myopia, macular thinning is often observed in the extrafoveal regions, whereas foveal thickness may conversely be increased,^[12]^ with a reduction in vascular density hypothesized to be associated with inner retinal thinning.^[13,14]^

Despite these global insights, a significant gap remains regarding the Japanese population. Most existing studies have either focused on a single modality (OCT or OCTA) or utilized cohorts that excluded specific age groups or eyes with significant refractive errors. In particular, given the high prevalence of myopia in East Asia, there is an urgent need for an inclusive analysis that simultaneously evaluates the combined influence of age, sex, and a wide range of axial lengths on both retinal structure and microcirculation. To our knowledge, such a systematic investigation has not yet been conducted in a diverse Japanese cohort of normal eyes. The purpose of this study was to investigate the combined effects of age, sex, refractive error, and a wide range of axial lengths on macular structure and perfusion in normal Japanese eyes using both OCT and OCTA.

## 2. Subjects and methods

### 2.1. Study design and participants

This retrospective, cross-sectional study included 297 adult participants (297 eyes; 136 male, 161 female; mean age ± standard deviation, 59.2 ± 14.4 years) presenting to Teine Keijinkai Hospital’s ophthalmology department between May 2019 and April 2023 (Table 1). Inclusion criteria were adults aged 20 years or older with no reported ocular or systemic symptoms and clinically normal eyes. Exclusion criteria included ocular surgery history, ocular disease other than mild cataract, suspected age-related macular degeneration or drusen, insufficient OCT image quality (oculomotor issues, anterior segment opacity, and poor fixation), diabetes mellitus, or uncontrolled hypertension. To ensure statistical independence and avoid bias associated with inter-eye correlation, only 1 eye per subject was included in the analysis. Generally, the right eye was selected; however, the left eye was used if the right eye did not meet the inclusion criteria or if its image quality was insufficient.

**Table 1:** Characteristics of study subjects.

Variables	Number or mean ± SD (range)
Number of subjects, n	297
Number of eyes, n	297
Age (yr)	59.2 ± 14.4 (18–86)
Gender, male/female	136/161
BCVA–logMAR	−-0.03 ± 0.11
IOP (mm Hg)	14.7 ± 2.7
SE, diopters	−1.98 ± 3.45 (+4.50 to −16.50)
AL (mm)	24.81 ± 1.70 (20.12–30.14)

AL = axial length, BCVA = best-corrected visual acuity; logMAR, logarithm of minimal angle of resolution, IOP = intraocular pressure, SD = standard deviation, SE = spherical equivalent.

### 2.2. Ethical statement

The Institutional Review Board of Teine Keijinkai Hospital approved this study (IRB number: 2-023313-00) with a waiver of informed consent (**opt-out method**). All procedures were conducted in accordance with the Declaration of Helsinki.

### 2.3. Ophthalmic examinations

All participants had comprehensive ophthalmic exams, including slit-lamp biomicroscopy and dilated fundus examination. Refraction and keratometry (autorefractometer ARK-1s, NIDEK), and intraocular pressure (noncontact tonometer TX-20P, Canon) were measured. Axial length was measured (IOLMaster 700, Carl Zeiss Meditec). Best-corrected visual acuity was converted to logMAR for analysis.

### 2.4. OCT and OCTA imaging

**Figure 1.**
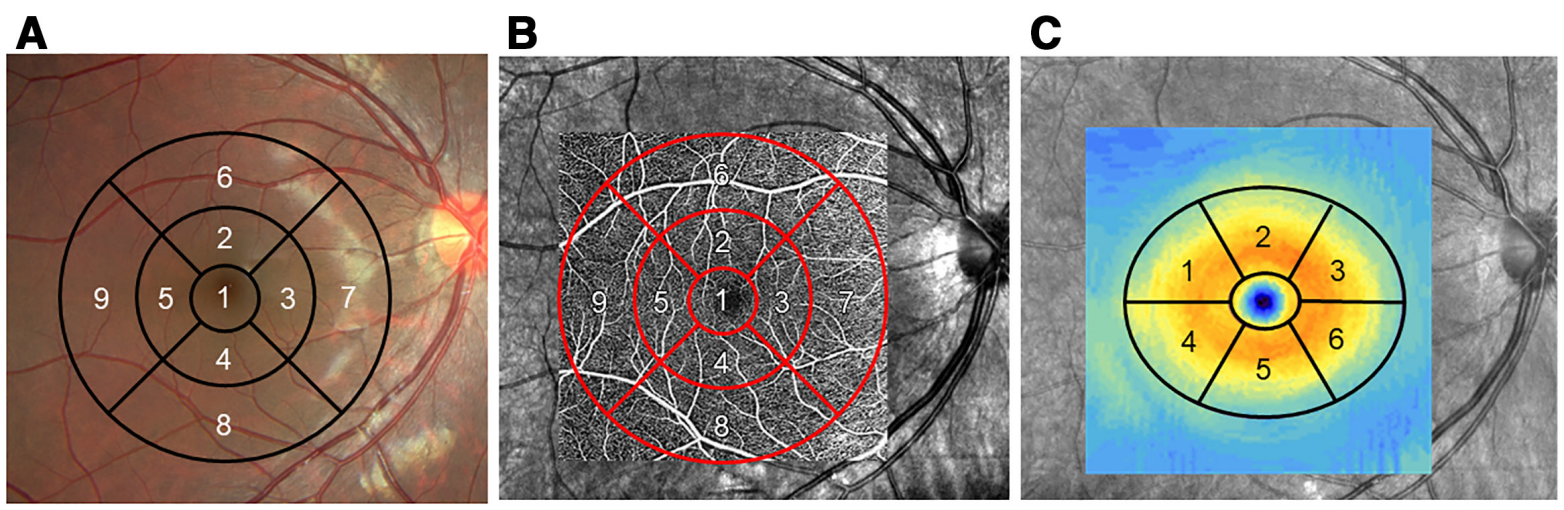
Retinal thickness and perfusion density in the macular area using OCT/OCTA. Macular thickness (A) and perfusion density (B) were assessed in 9 sectors by the Early Treatment Diabetic Retinopathy Study (ETDRS) grid. 1: central, 2: inner ring superior, 3: inner ring nasal, 4: inner ring inferior, 5: inner ring temporal, 6: outer ring superior, 7: outer ring nasal, 8: outer ring Inferior, 9: outer ring temporal. Macular ganglion cell-inner plexiform layer (GCIPL) thickness (*C*) was evaluated in 6 sectors of the annular region between a 1 × 1.2 mm circle and a 4 mm × 4.8 mm circle. 1: superotemporal, 2: superior, 3: supreronasal, 4: inferotemporal, 5: inferior, 6: inferonasal. OCT = optical coherence tomography, OCTA = optical coherence tomography angiography


Macular OCT and OCTA images were acquired using a Cirrus HD-OCT 5000 (Carl Zeiss Meditec) after pupillary dilation (0.5% tropicamide and 0.5% phenylephrine). Macular (6 × 6 mm) retinal thickness and superficial capillary plexus (SCP) perfusion density were automatically quantified (built-in software) on an Early Treatment Diabetic Retinopathy Study (ETDRS) grid (9 sectors, Fig. 1A). Perfusion density was defined as perfused vessel area per unit area. SCP OCTA images used the device’s algorithm (Fig. 1B). The SCP slab was inner limiting membrane (upper) to approximated inner plexiform layer (lower), calculated as:

$${\text{Z}}_{\text{I}PL}={\text{Z}}_{\text{I}LM}+70\%\times {\text{T}}_{\text{I}LM}{-}_{\text{O}PL},$$ (1)

where *Z*, *T*, and OPL are boundary location, thickness, and outer plexiform layer. Ganglion cell-inner plexiform layer (GCIPL) thickness was automatically measured in the 1 × 1.2 mm to 4 × 4.8 mm macular annulus (built-in ganglion cell analysis algorithm, Fig. 1C). Only scans with OCT signal ≥ 7 and OCTA ≥ 8 were included.


ZIPL=ZILM+70%×TILM−OPL (2)

### 2.5. Statistical analysis

Descriptive statistics (means and standard deviations) were calculated for all primary outcomes. Multiple regression analyses investigated associations between retinal thickness and perfusion density (dependent variables) and age, sex (male = 0, female = 1), refractive error, and axial length (independent variables). Statistical significance was set at *P* < .05. Standardized regression coefficients (β) were reported to assess each independent variable’s relative contribution. To ensure sufficient statistical power, an a priori power analysis was performed based on effect sizes reported in previous literature.^[4]^ For the comparison of retinal thickness between sexes, the required sample size to achieve a power of 0.80 at an alpha level of 0.05 was determined to be 90 eyes. Our sample size of 297 eyes provided more than sufficient capacity to detect significant associations. SPSS version 21 (IBM Corporation,) was used for all statistical analyses.

## 3. Results

### 3.1. General characteristics of macular parameters

Mean retinal thickness across the 9 ETDRS grid regions was 271 ± 13 μm, with a mean perfusion density of 43.7% ± 3.2% (Table 2). In the central subfield, these values were 253 ± 20 μm and 20.2% ± 6.1%, respectively. Retinal thickness exhibited a nasal–temporal gradient in both inner and outer rings, with nasal regions being thicker. Mean GCIPL thickness was 79 ± 6 μm (Table 2).

**Table 2:** Macular retinal thickness and perfusion density in the ETDRS grid, and sectoral GCIPL thickness in normal eyes.

Subfield	Retinal thickness (μm)	Perfusion density (%)	GCIPL thickness (μm)
Central (1 mm)	253 ± 20	20.2 ± 6.1	
Inner ring (3 mm)			
Superior	319 ± 15	44.2 ± 3.2	
Nasal	322 ± 16	42.4 ± 4.0	
Inferior	315 ± 15	43.0 ± 3.7	
Temporal	308 ± 15	43.7 ± 3.4	
Outer ring (6 mm)			
Superior	274 ± 14	45.5 ± 3.6	
Nasal	294 ± 15	48.3 ± 2.9	
Inferior	261 ± 14	44.5 ± 4.2	
Temporal	257 ± 13	40.4 ± 5.5	
Whole sector	271 ± 13	43.7 ± 3.2	79 ± 6
Superotemporal	–	–	79 ± 6
Superior	–	–	80 ± 6
Supreronasal	–	–	81 ± 6
Inferotemporal	–	–	80 ± 6
Inferior	–	–	77 ± 7
Inferonasal	–	–	79 ± 7

ETDRS = Early Treatment Diabetic Retinopathy Study, GCIPL = ganglion cell-inner plexiform layer.

### 3.2. Factors associated with retinal thickness

Multiple regression analysis revealed significant associations between retinal thickness in ETDRS regions and age, sex, and axial length (Table 3). Older age was associated with retinal thinning in 8 regions excluding the central region (*P* < .05). Female sex was associated with thinner retinas in all regions (*P* < .05). Refractive error showed no significant association with retinal thickness. Longer axial length was associated with thinning in the same regions affected by age (*P* < .05), but showed increased thickness in the central region (*P*=.101).

**Table 3:** Multiple regression analysis of age, refractive power, and axial length for macular retinal thickness of normal eyes in the ETDRS grid.

Subfield	Age	*P* value	Sex	*P* valuee	SE	*P* value	AL	*P* value	*R* ^2^
	β		β		β		β		
Central (1 mm)	−0.10	.101	**−0.22**	**<.001**	0.09	.385	**0.27**	**.013**	0.16
Inner ring (3 mm)									
Superior	**−0.13**	**.038**	**−0.27**	**<.001**	−0.11	.280	**−0.28**	**.013**	0.06
Nasal	**−0.16**	**.010**	**−0.32**	**<.001**	−0.02	.818	−0.17	**<.001**	0.10
Inferior	**−0.13**	**.036**	**−0.30**	**<.001**	−0.08	.445	**−0.32**	**.005**	0.08
Temporal	−0.10	.114	**−0.33**	**<.001**	−0.08	.433	**−0.22**	**<.001**	0.08
Outer ring (6 mm)									
Superior	**−0.25**	**<.001**	**−0.17**	**.001**	**−0.21**	**.004**	**−0.60**	**<.001**	0.14
Nasal	**−0.28**	**<.001**	**−0.17**	**.007**	−0.12	.239	**−0.40**	**<.001**	0.09
Inferior	**−0.24**	**<.001**	**−0.19**	**.002**	−0.10	.308	**−0.56**	**<.001**	0.15
Temporal	**−0.14**	**.018**	**−0.25**	**<.001**	−0.08	.399	**−0.53**	**<.001**	0.15

Bold values indicate statistically significant associations.

AL = axial length, ETDRS = Early Treatment Diabetic Retinopathy Study, SE = spherical equivalent.

### 3.3. Factors associated with GCIPL thickness

Multiple regression analysis also assessed GCIPL thickness relationships with age, sex, and axial length (Table 4). Older age was associated with GCIPL thinning in all regions (*P* < .001). Sex was associated with GCIPL thickness in the superotemporal and inferotemporal regions (*P* < .05). No significant association was found between GCIPL thickness and refractive error. Longer axial length was significantly associated with decreased GCIPL thickness (*P* < .001).

**Table 4:** Multiple regression analysis of age, gender, refractive power, and axial length for sectoral GCIPL thickness of normal eyes.

Subfield	Age	*P* value	Sex	*P* value	SE	*P* value	AL	*P* value	*R* ^2^
	β		β		β		β		
Superotemporal	**−0.29**	**<.001**	**−0.15**	**.016**	−0.11	.241	**−0.54**	**<.001**	0.14
Superior	**−0.33**	**<.001**	−0.09	.122	−0.11	.228	**−0.66**	**<.001**	0.24
Supreronasal	**−0.33**	**<.001**	−0.07	.183	−0.04	.645	**−0.65**	**<.001**	0.28
Inferotemporal	**−0.28**	**<.001**	**−0.12**	**.048**	−0.11	.283	**−0.58**	**<.001**	0.17
Inferior	**−0.25**	**<.001**	−0.06	.284	−0.13	.184	**−0.68**	**<.001**	0.25
Inferonasal	**−0.32**	**<.001**	−0.04	.513	−0.00	.982	**−0.60**	**<.001**	0.26

Bold values indicate statistically significant associations.

AL = axial length, GCIPL = ganglion cell-inner plexiform layer, SE = spherical equivalent.

### 3.4. Factors associated with perfusion density

Finally, multiple regression examined perfusion density relationships in ETDRS regions with age, sex, and axial length (Table 5). Older age was associated with decreased perfusion density in all regions (*P* < .05). No significant association was found between perfusion density and sex or refractive error. Longer axial length was significantly associated with decreased perfusion density in the outer ring superior, nasal, inferior, and temporal regions (*P* < .01).

**Table 5:** Multiple regression analysis of age, refractive power, and axial length for macular perfusion density of normal eyes in the ETDRS grid.

Subfield	Age	*P* value	Sex	*P* value	SE	*P* value	AL	*P* value	*R* ^2^
	β		β		β		β		
Central (1 mm)	**−0.30**	**<.001**	–0.05	.435	0.18	.087	0.20	.072	0.11
Inner ring (3 mm)									
Superior	**−0.20**	**.002**	–0.06	.331	0.04	.692	–0.19	**<.001**	0.04
Nasal	**−0.16**	**.016**	–0.07	.310	–0.03	.788	–0.25	**.033**	0.03
Inferior	**−0.18**	**.006**	–0.02	.792	0.04	.702	–0.15	.191	0.02
Temporal	**−0.15**	**.002**	–0.02	.735	0.09	.400	–0.21	.070	0.05
Outer ring (6 mm)									
Superior	**−0.42**	**<.001**	–0.02	.710	–0.11	.281	**−0.53**	**<.001**	0.20
Nasal	**−0.33**	**<.001**	–0.06	.320	–0.16	.118	**−0.26**	**.020**	0.10
Inferior	**−0.40**	**<.001**	–0.07	.221	0.02	.845	**−0.43**	**<.001**	0.18
Temporal	**−0.31**	**<.001**	–0.09	.135	0.15	.115	**−0.32**	**<.001**	0.16

Bold values indicate statistically significant associations.

AL = axial length, ETDRS = Early Treatment Diabetic Retinopathy Study, SE = spherical equivalent.

### 3.5. Model performance and multicollinearity

The coefficient of determination (*R*^2^) ranged from 0.06 to 0.28, indicating low to moderate predictive accuracy. The variance inflation factor was low for age, sex, refractive error, and axial length (1.26, 1.25, 3.44, and 3.94, respectively), indicating no clinically significant multicollinearity among the independent variables.

## 4. Discussion

This study provides novel insights into the relationship between macular structure and demographic/ocular characteristics in normal Japanese subjects. Using OCT and OCTA, we quantified retinal thickness and SCP perfusion density across a wide age range and found significant associations with age, sex, and axial length, but not with refractive error. Notably, both retinal thickness and perfusion density tended to decrease with age and axial length, with relative sparing of the central macula. We also observed consistently thinner retinas in female subjects.

### 4.1. Age

Aging is a complex, irreversible process marked by a time-dependent decline in physiological function.^[1,15–17]^ Numerous OCT studies have investigated age-related retinal structural changes, consistently showing decreased thickness in the inner, outer, and total retina.^[2,3,8]^ This aligns with age-related neuronal loss, documented by histological analysis of retinal ganglion cells/axons,^[9]^ and optical measurements of the retinal nerve fiber layer.^[18,19]^ Retinal nerve fiber layer thinning is estimated at ~0.2%/yr.^[10]^

Our study confirmed a negative correlation between age and inner retinal layer thickness (ganglion cell layer to outer inner plexiform layer border) across all macular sectors, stronger than for total retinal thickness. Consistent with Ooto et al,^[4]^ we found no significant age-related decrease in foveal thickness, unlike inner retinal layer thinning. This is supported by Curcio et al,^[11]^ who reported a 30% rod density reduction (ages 27–90) with no consistent age-related cone density change. This small photoreceptor number change likely minimally impacts photoreceptor layer thickness, explaining our OCT findings of minimal foveal thinning with age.

The effect of age on retinal pigment epithelium (RPE) thickness is less clear. Some report age-related RPE/Bruch membrane thickening,^[20]^ others thinning after 45.^[21]^ As Cirrus HD-OCT 5000 uses the mid-RPE layer as the outer retinal boundary,^[22]^ our study may not have adequately captured age-related RPE changes, requiring further investigation.

Consistent with recent research, our study also provides data on age-related macular capillary density changes. Several studies show a negative association between aging and parafoveal vascular density in both SCP and deep capillary plexus (DCP).^[5–7,23]^ Wei et al^[24]^ demonstrated concurrent inner retinal layer thinning and retinal vessel changes with age (18–82).

The retinal thinning and hypoperfusion observed are consistent with these reports and likely reflect normal aging. However, the precise causal link between microvascular decline and age-related neurodegeneration is unclear. It is uncertain if microvascular network decline is due to age-related vascular damage or a response to reduced metabolic demand from degenerating neurons. Overall, these findings support a model where multiple retinal components are affected by aging, suggesting a complex interplay of structural and vascular changes.

### 4.2. Sex

Prior studies consistently show a sex-related effect on macular thickness, with females generally exhibiting significantly thinner retinas than males.^[4,5,8]^ Our findings corroborate these observations, showing significantly reduced retinal thickness in females across all ETDRS subfields. Importantly, this difference remained significant even after adjusting for axial length, suggesting that the thinner retina in females is an intrinsic anatomical feature rather than a secondary effect of ocular size. This difference may relate to the higher prevalence of idiopathic macular holes in females, particularly over 60,^[25,26]^ although the precise link between macular thinning and IMH pathogenesis is unclear.

Literature on sex-related differences in vascular density is less consistent. Coscas et al^[23]^ reported higher vascular density in females over 60, suggesting potentially slower vascular aging. Conversely, Wang et al^[7]^ found higher vascular density in males. Consistent with our findings, several studies report no significant association between parafoveal vascular density and sex.^[6,27]^ Thus, while our study confirms decreased macular thickness in females, we found no significant sex differences in intramacular perfusion density. These structural macular variations between sexes may improve understanding of sex-related differences in visual function and ocular health.

Recent research explores artificial intelligence for sex identification via retinal image analysis.^[28]^ Studies using color fundus photographs identified sex-related features like the papillomacular angle, retinal vessel angles, and artery trajectory.^[29,30]^ While deep learning models showed promise in predicting sex from color fundus photographs, central foveal pathology reduces model performance, highlighting the fovea’s importance in sex prediction.^[30]^ However, the specific pathologies and their severity are not fully characterized. Despite growing research using retinal images for sex prediction, few studies explicitly integrate data on distinct retinal features observed between sexes. Further research is needed to elucidate the biological mechanisms underlying these sex-related differences.

### 4.3. Axial length

Excessive ocular axial elongation is a key pathogenic mechanism in myopia. In myopic eyes, increased scleral surface area stretches and thins the choroid and retina, potentially leading to complications.^[31]^ Shimada et al^[32]^ demonstrated retinal blood vessel constriction and reduced flow in highly myopic eyes.

Numerous OCT studies correlate macular thickness and axial length. A meta-analysis^[33]^ showed significantly lower mean retinal thickness in highly myopic eyes versus controls, particularly in perifoveal and parafoveal regions. Similar ganglion cell complex thinning occurs in moderate/high myopia. Consistent with this, our study confirmed significant GCIPL thickness decrease with increasing axial length, suggesting elongation is associated with increased retinal surface area and decreased ganglion cell density.

Conversely, we observed increased foveal retinal thickness with longer axial length. Several hypotheses exist, including tangential traction involving the posterior vitreous cortex or internal limiting membrane.^[34]^ While some suggest increased foveal thickness could be influenced by ocular magnification artifacts,^[12]^ Kim et al reported similar results even after adjusting for magnification.^[13]^ Our observation of this topographical disparity – where the fovea remains spared or thickened while the outer regions thin – aligns with these established anatomical and clinical findings, suggesting that these changes reflect genuine structural variations associated with axial elongation.^[14]^

Several studies document decreased retinal microvessel density in myopic maculae. In our study, ETDRS outer ring SCP perfusion density significantly decreased with increasing axial length, consistent with lower parafoveal perfusion in high myopia.^[35,36]^ Khan et al^[37]^ linked inner retinal layer thinning to reduced vessel density, a potential pathway to ganglion cell loss. Interestingly, we found a positive correlation between central foveal SCP perfusion density and axial length, possibly related to increased foveal retinal thickness. Jonas et al^[14]^ showed retinal thickness decreases with axial length in the equatorial/preequatorial fundus, while macular thickness is minimally affected. These results further elucidate axial elongation’s effects, where the fovea and outer rings exhibit distinct, independent responses to globe expansion.

Notably, axial length showed significant associations with macular parameters whereas refractive error did not. This highlights axial length as a more direct structural biomarker of physical globe stretching, rather than refractive error, which is a functional composite of multiple optical factors. Supported by low variance inflation factor values, our results suggest that axial length should be prioritized over refractive error for the accurate interpretation of OCT and OCTA data in clinical practice.

These findings have important implications for understanding the complex relationship between ocular axial elongation and visual function.

### 4.4. Clinical implications and summary

In summary, our findings demonstrate that retinal structure and microcirculation in normal Japanese eyes are regulated by age, sex, and axial length in a complex and region-specific manner. The contrasting patterns observed – namely, the thinning and reduced perfusion in the outer macular regions associated with aging and axial elongation, versus the relative sparing or paradoxical increase in the fovea – highlight the unique anatomical and physiological resilience of the central macula.

Understanding the magnitude of these physiological variations is paramount for the early and accurate identification of pathological changes. Relying on universal normative databases without considering individual demographic and ocular characteristics may lead to misinterpretation of data. Therefore, we propose that an individualized diagnostic approach, incorporating adjustments for age, sex, and axial length, is essential for enhancing the precision of OCT and OCTA in both clinical research and daily practice.

### 4.5. Limitation

This study has several limitations. First, its retrospective design may have introduced selection bias. Future prospective studies with robust designs are needed. Second, our OCTA measurements were limited to the SCP. Similar or more pronounced effects may exist in the DCP, warranting further investigation with DCP-capable OCTA devices. Third, GCIPL and OCTA scanned regions were not perfectly congruent, potentially influencing interpretation. Finally, axial length-based magnification correction was not performed. While this lack of correction may particularly affect the absolute values in eyes with longer axial length, our finding of distinct, opposing topographical patterns between the fovea and outer regions aligns with results from previous corrected models. Therefore, the observed directional associations likely reflect genuine anatomical trends, though future studies with rigorous magnification adjustment are warranted.

## 5. Conclusion

This study highlights the significant impact of age, sex, and axial length on quantitative OCT and OCTA metrics in normal eyes. Our findings suggest that an individualized approach, rather than reliance on single normative values, is essential for the accurate and early detection of ocular diseases.

## Author contributions

**Conceptualization:** Yuki Ito, Hiroaki Endo.

**Data curation:** Yuki Ito, Hiroaki Endo.

**Formal analysis:** Yuki Ito, Hiroaki Endo.

**Investigation:** Yuki Ito, Hiroaki Endo, Natsuko Shichinohe.

**Methodology:** Yuki Ito, Hiroaki Endo.

**Project administration:** Hiroaki Endo.

**Supervision:** Satoru Kase, Natsuko Shichinohe, Mitsuo Takahashi, Manabu Kase.

**Validation:** Satoru Kase, Natsuko Shichinohe, Mitsuo Takahashi, Manabu Kase.

**Visualization:** Yuki Ito, Hiroaki Endo.

**Writing – original draft:** Yuki Ito, Hiroaki Endo, Satoru Kase, Manabu Kase.

**Writing – review & editing:** Yuki Ito, Hiroaki Endo, Satoru Kase, Manabu Kase.
